# Selective loss of glucocerebrosidase activity in sporadic Parkinson’s disease and dementia with Lewy bodies

**DOI:** 10.1186/s13024-015-0010-2

**Published:** 2015-03-27

**Authors:** Davide Chiasserini, Silvia Paciotti, Paolo Eusebi, Emanuele Persichetti, Anna Tasegian, Marzena Kurzawa-Akanbi, Patrick F Chinnery, Christopher M Morris, Paolo Calabresi, Lucilla Parnetti, Tommaso Beccari

**Affiliations:** Dipartimento di Medicina, Sezione di Neurologia, Università degli Studi di Perugia, Sant’ Andrea delle Fratte, 06132 Perugia, Italy; Department of Pharmaceutical Sciences, University of Perugia, Via Fabretti 48, 06123 Perugia, Italy; Health Planning Service, Regional Health Authority of Umbria, 06124 Perugia, Italy; Wellcome Centre for Mitochondrial Research, Newcastle University, Newcastle upon Tyne, UK; Institute of Genetic Medicine, Newcastle University, Newcastle upon Tyne, UK; Institute of Neuroscience, Medical Toxicology Centre and NIHR Health Protection Research Unit in Chemical and Radiation Threats and Hazards, Wolfson Building, Newcastle University, Claremont Place, Newcastle upon Tyne, NE2 4AA UK; Fondazione S. Lucia, IRCCS, Rome, Italy

**Keywords:** Parkinson’s disease, Lysosome, β-glucocerebrosidase, β-hexosaminidase, Substantia nigra

## Abstract

**Background:**

Lysosomal dysfunction is thought to be a prominent feature in the pathogenetic events leading to Parkinson’s disease (PD). This view is supported by the evidence that mutations in *GBA* gene, coding the lysosomal hydrolase β-glucocerebrosidase (GCase), are a common genetic risk factor for PD. Recently, GCase activity has been shown to be decreased in substantia nigra and in cerebrospinal fluid of patients diagnosed with PD or dementia with Lewy Bodies (DLB). Here we measured the activity of GCase and other endo-lysosomal enzymes in different brain regions (frontal cortex, caudate, hippocampus, substantia nigra, cerebellum) from PD (n = 26), DLB (n = 16) and age-matched control (n = 13) subjects, screened for *GBA* mutations. The relative changes in GCase gene expression in substantia nigra were also quantified by real-time PCR. The role of potential confounders (age, sex and post-mortem delay) was also determined.

**Findings:**

Substantia nigra showed a high activity level for almost all the lysosomal enzymes assessed. GCase activity was significantly decreased in the caudate (−23%) and substantia nigra (−12%) of the PD group; the same trend was observed in DLB. In both groups, a decrease in GCase mRNA was documented in substantia nigra. No other lysosomal hydrolase defects were determined.

**Conclusion:**

The high level of lysosomal enzymes activity observed in substantia nigra, together with the selective reduction of GCase in PD and DLB patients, further support the link between lysosomal dysfunction and PD pathogenesis, favoring the possible role of GCase as biomarker of synucleinopathy. Mapping the lysosomal enzyme activities across different brain areas can further contribute to the understanding of the role of lysosomal derangement in PD and other synucleinopathies.

**Electronic supplementary material:**

The online version of this article (doi:10.1186/s13024-015-0010-2) contains supplementary material, which is available to authorized users.

## Introduction

The identification of the underlying causes of Parkinson’s disease (PD) is a major challenge, since in most instances, cases present with sporadic disease. The discovery of the genetic determinants of PD would be a major step forward in describing the underlying etiology of the disorder and also in identifying possible therapeutic strategies [1]. Mutations in the *GBA* gene, encoding for the lysosomal hydrolase β-glucocerebrosidase (GCase, EC = 3.2.1.45) cause Gaucher disease (GD) a rare lysosomal storage disorder, and represent a common risk factor for PD [2-4]. Patients carrying *GBA* loss of function mutations have a five-fold increased risk of developing PD with respect to non-carriers [3], and may show a similar phenotype to idiopathic PD. The GCase substrate, glucosylceramide, stabilizes soluble oligomeric α-syn species [5]. As a consequence, GCase deficiency may contribute to α-syn aggregation and accumulation. GCase deficiency has been identified in post mortem brain from patients diagnosed with PD either with or without *GBA* mutations, the decrease being most evident in the substantia nigra, cerebellum and cortex of PD patients [6,7]. This non-selective loss of GCase activity irrespective of mutation status may either represent a global defect in lysosomal enzymes in PD, or may simply represent the presence of other pathologies such as neuronal loss leading to deficiency.

The activity of GCase and other lysosomal enzymes can also be reliably measured in cerebrospinal fluid (CSF) [8]. The reduction of GCase activity has been found also in the cerebrospinal fluid (CSF) of PD patients when compared to neurological controls [9-11] and in fibroblasts from patients with GD or PD carrying *GBA* mutations [12]. Interestingly, other lysosomal enzymes involved in different degradation pathways, were also found to be altered in PD patients diagnosed with PD. An increased β-hexosaminidase activity, a lysosomal enzyme able to hydrolyze the GM2 ganglioside, has been found in CSF and fibroblasts of PD patients [11,12]. Another study showed increased activity of cathepsin E and β-galactosidase in CSF of de-novo PD patients while α-fucosidase activity was significantly decreased [13]. Together, these findings suggest that there may be a more widespread dysfunction of lysosomal enzymes in PD and related disorders.

Here we determined the specific activity of several endo-lysosomal enzymes, namely β-hexosaminidase, α-fucosidase, β-mannosidase, α-mannosidase, β-galactosidase, β-glucocerebrosidase and cathepsin E, in different brain areas of PD patients, age-matched controls and, for some brain areas, of patients diagnosed with dementia with Lewy bodies (DLB). Our aims were to map the activity of these lysosomal enzymes in different human brain regions and also to evaluate the occurrence of lysosomal dysfunction not only in PD but also in another synucleinopathy, DLB. This would provide the necessary information to determine if lysosomal dysfunction is widespread in PD, or if there is a relatively selective loss of GCase.

## Findings

**Figure 1:**
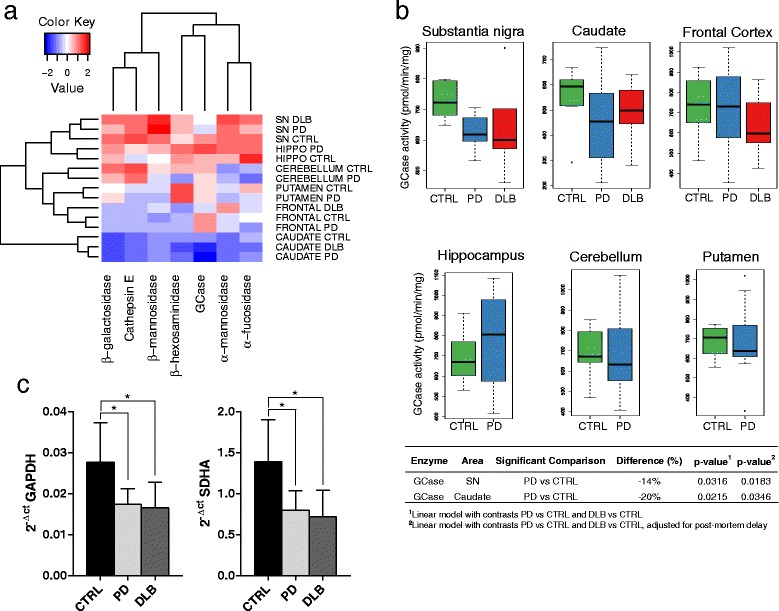
**Lysosomal enzyme activities in human brain. a)** Cluster analysis of lysosomal enzyme activities. Lysosomal specific activities were normalized with respect to their means and standard deviations, while patients were summarized using their respective diagnostic group; **b)** Boxplots of the GCase specific activity across different brain areas. In the table below the figure, the significant comparisons are reported according to the statistical model used; **c)** mRNA levels of GBA gene. 2^-ΔCT^ values normalized by housekeeping genes GAPDH and SDHA. *p < 0.05 using Wilcoxon two groups test and nonparametric ANCOVA model with adjustment for post mortem delay.

We utilized a large series of clinically and neuropathologically verified cases of PD and DLB and age matched control cases. In Table 1 the demographic characteristics of the patients included in the study are reported. Post-mortem brain tissue was analyzed for lysosomal enzyme activity, GBA genotype and GBA mRNA expression. GBA sequencing was performed to verify the presence of pathological mutations on GBA gene. The results showed that our cohort was composed mainly of patients and controls without GBA mutations. Only two out of 26 PD patients were heterozygous for pathogenic GBA mutations. One patient carried the L444P mutation while the other one carried the IVS2 + 1G > A mutation. The remaining PD and DLB patients and control subjects were wild type for the GBA gene (CTRL =9, PD = 23, DLB = 15). GBA genotype was not available in 4 controls, 1 PD and 1 DLB. Using hierarchical clustering analysis, we compared the lysosomal enzyme activities across different brain areas (Figure 1a). The substantia nigra and the hippocampus clustered together in the analysis, globally showing a high level of almost all the enzyme activities. GCase was the exception, showing low levels in substantia nigra but only in the pathological groups, while its levels were high in frontal cortex. The caudate clustered independently from the other areas and showed the lowest levels of the measured activities. Notably, the differences among the brain areas were greater than the differences between PD, DLB patients and control subjects.

**Table 1:** **Demographic characteristics of the subjects included in the study**

**Demographics**	**Controls**	**PD**	**DLB**
N	13	26	16
Sex (M)	6 (46%)	18 (69%)	12 (75%)
Age	77.0 (±8.0), 78 (65–89)	76.4 (±6.4), 77 (64–89)	76.9 (±5.7), 77 (66–88)
Post mortem delay	18.2 (±6.9), 17 (8–30)	26.2 (±12.8), 24 (7–64)*	18.5 (±8.6), 18 (4–31)
Alzheimer Braak stage	1.9 (±1.4), 2 (0–4)	2.1 (±1.5), 2 (0–5)*	2.6 (±1.1), 2.5 (0–4)

Means (±SD), medians (min-max). *p < 0.05 with Wilcoxon two groups test or Fisher’s exact test. Braak stage is referred to the Alzheimer’s neurofibrillary tangle score.

The comparison of the mean levels of lysosomal enzyme activities in the three groups is reported in Additional file 1: Table S1. We first evaluated the effect of post mortem delay as a confounder in the different comparisons. No significant association was found between postmortem delay and lysosomal enzymes activity when it was considered as a potential confounder (data not shown). GCase specific activity in substantia nigra and caudate was significantly lower in PD patients when compared to control subjects (p < 0.01 and p < 0.05 respectively, Figure 1b). In DLB patients, GCase activity was also reduced, but the difference was not significant (Additional file 1: Table S1). GCase activity did not change significantly in the other brain regions in any of the diagnostic groups (Figure 1b), suggesting a selective loss in the nigrostriatal system. The two patients carrying *GBA* mutations showed detectable GCase activity, with values within the range of the PD and DLB patients without mutations. Exclusion of the two patients from the analysis did not change the significance of the final results (data not shown).

To evaluate if the decrease in GCase activity was due to a change in gene expression we performed qPCR on *GBA* mRNA in the substantia nigra. mRNA levels of *GBA* gene, normalized against GAPDH, were significantly lower in PD and DLB compared to controls (p < 0.05). The same result was found with respect to the subunit A of the succinate dehydrogenase complex (SDHA) (Figure 1c).

Other enzymatic activities were changed in different brain areas. In particular, α-fucosidase activity in frontal cortex was significantly lower in PD compared to controls (p < 0.01), while its activity in DLB patients did not change significantly (Additional file 1: Figure S1). Interestingly, α-mannosidase activity was increased in frontal cortex only in DLB patients (Additional file 1: Figure S1).

We also performed correlation analysis between the enzyme activities and Alzheimer’s neurofibrillary pathology expressed as Braak stage. No significant associations were found for any of the enzyme activities tested (data not shown).

## Discussion

GCase is recognized as an important risk factor for PD and DLB. A recent multicenter study found a more significant association of *GBA* mutations with DLB compared to PD with dementia [14]. In this study we mapped the activity of a series of endo-lysosomal enzymes in post mortem brain tissue of PD and DLB patients, and compared these with elderly pathologically normal control brains from patients. We confirm that GCase specific activity is reduced in PD brains, especially in substantia nigra and that this reduction was also present in DLB patients although to a lesser extent. Interestingly, the expression of GCase at mRNA level was also reduced in the substantia nigra of both synucleinopathies. Our results confirm previous reports on the deregulation of GCase in PD [6,7]. In PD patients carrying *GBA* mutations, GCase activity has been found to be reduced in all the brain areas analyzed with exception of the cortex [6]. Recently, in sporadic PD, GCase activity and protein expression were found to be decreased in the anterior cingulate and occipital cortex, where accumulation of α-synuclein occurs during the early phases of the disease [7]. GCase activity decrease in these two regions was associated with a reduced lysosomal chaperone-mediated autophagy and decreased ceramide levels [7].

It is worth noting that in the study of Gegg et al. [6] mRNA levels of GCase were unchanged, while in Murphy et al. [7] GCase expression was reduced. In our study, measuring GCase mRNA expression in substantia nigra of PD and DLB patients, we also identified a significant reduction of GCase mRNA in both PD and DLB. This decrease was paralleled by a significant reduction of GCase activity (PD > DLB). Previous work on DLB brains also showed a trend in reduction of GCase activity in patients without *GBA* mutation, while in *GBA* mutations carriers the activity of the enzyme was drastically reduced with respect to control subjects [15]. In this study, the low number of patients carrying heterozygous *GBA* mutations prevented us from finding any significant relationship between *GBA* genotype and GCase activity. Nevertheless, we cannot exclude that heterozygous *GBA* mutations might contribute to the reduction of GCase activity in PD.

With respect to the other lysosomal enzymes considered in this study, we found a significant reduction of α-fucosidase activity in the frontal cortex of PD patients compared to control subjects, while in DLB the reduction was not significant. α-Fucosidase is responsible for the hydrolysis of α-1,6-fucose residues from glycoproteins and has been previously found to be reduced in CSF from de-novo PD patients [13]. The possible involvement of α-fucosidase in PD pathogenesis is currently unknown, however brain protein fucosylation is regarded as an important way for regulating different processes such as neurite outgrowth and synaptic plasticity [16]. Defective degradation of fucose residues in proteins may contribute to a deregulation of these processes and/or accumulation of undegraded substrates in lysosomes.

In a study by McNeil and colleagues other lysosomal enzymes were altered in fibroblast from PD patients with *GBA* mutation [12]. Cathepsin D and β-hexosaminidase showed increased activity in PD patients fibroblasts, possibly supporting a global deregulation of lysosome functioning. In the present study, the activity of β-hexosaminidase did not show significant change in any of the brain regions assessed in the three experimental groups. Overall, however, we identified minimal changes in other lysosomal enzymes in PD or DLB, suggesting that the defect in GCase may be selective in PD and DLB.

Globally, we found that the activity of the lysosomal enzymes tested was higher in substantia nigra and hippocampus with respect to the other brain areas. This result is particularly interesting because it highlights the important role of the lysosome system in the metabolism of substantia nigra. It might be possible that, in this region, lysosomal metabolism is more active due to the elevated oxidative stress taking place in this brain area, with the production of oxidized proteins and lipids [17,18]. A failure of the lysosomal system may trigger the accumulation of specific substrates of lysosomal hydrolases and proteases, including α-synuclein, as shown in animal and cell models of GCase inhibition [19,20].

In conclusion, we have mapped the lysosomal enzyme activity in two types of synucleinopathies. The GCase deficiency found in both diseases and specifically present in PD at two different regulation levels (i.e. mRNA and activity) supports the hypothesis of a common mechanism of GCase reduction in the pathogenesis of synucleinopathies, with a strong link to lysosome dysfunction.

## Methods

All aspects of this study were approved through the local Ethics Committee of the University of Perugia and the Newcastle upon Tyne Research Ethics Committee. Our study utilized 55 post-mortem brains, 26 diagnosed with PD, 16 with DLB and 13 controls without any neurodegenerative disease (age and gender matched). Brain tissue was obtained from the Newcastle Brain Tissue Resource (NBTR) at Newcastle University, UK, from individuals with a clinical diagnosis of Parkinson’s disease or dementia with Lewy bodies following informed consent from donors and with assent from the next of kin. Tissue from individuals without a history of cognitive impairment or movement disorder served as controls. Neuropathological assessment was performed according to standardized neuropathological diagnostic procedures [21] and confirmed the presence of Parkinson’s disease pathology and pathological changes of dementia with Lewy bodies, or an absence of significant neuropathology in control cases. For each patient, samples of snap frozen hippocampus, substantia nigra, putamen, frontal cortex and caudate were used. *GBA* genotyping was carried out according to Kurzawa-Akanbi and colleagues [15].

### Enzymatic assays

Brain tissues were thawed and lysed on ice in 50 mM phosphate buffer pH 7.0, containing 150 mM NaCl, 5% w/v, using an Ultra-turrax. 0.1% NP-40 detergent was added and homogenized tissues were sonicated for 30 seconds on ice at 20 Watt. The samples were kept on ice for 30 minutes and centrifuged for 10 minutes at 16,000xg and the supernatants were used for assay. The activities of the lysosomal enzymes β-hexosaminidase, α-fucosidase, β-mannosidase, α-mannosidase, β-galactosidase, β-glucorecebrosidase and cathepsin E were determined as previously described [8,11]. All of the activities were measured in triplicate. One unit (U) of enzyme activity was defined as the amount of enzyme that hydrolyses 1 pmol of substrate/min at 37°C.

### mRNA levels of GBA gene

Total RNA was extracted from substantia nigra tissue using standard methods and the relative changes in GBA gene expression were determined by qPCR via relative quantification using GAPDH and SDHA as housekeeping genes (Applied Biosystem 7300, TaqMan gene expression assay, probe numbers GBA: Hs00986836_g1, SDHA: Hs00188166_m1, GAPDH: Hs02758991_g1). The 2^-ΔCT^ method [22] was used to calculate relative changes in gene expression determined from the real-time quantitative PCR analysis.

### Data analysis

The R software [23] was used for statistical analyses. Continuous variables are presented as means (±standard deviations) and medians (ranges). Categorical variables are presented as count and percentages. Hierarchical clustering analysis was used to compare the lysosomal enzyme activities across different brain areas. Differences between patients and controls were assessed using ANOVA for both comparing lysosomal enzymes activities and *GBA* mRNA expression. Dunnett’s post-hoc test was used for multiple comparisons. ANCOVA was performed for testing the confounding effects of post-mortem delay on the relationships between lysosomal enzymes activity or *GBA* mRNA expressions and disease. A p-value ≤0.05 was considered significant in all the analyses.

## Additional file

Additional file 1: Figure S1. Boxplot of lysosomal enzyme activities significantly different among the experimental groups. **Table S1.** Lysosomal enzymes activities for brain areas and groups.

## References

[1] Singleton AB, Farrer MJ, Bonifati V (2013). The genetics of Parkinson’s disease: progress and therapeutic implications. Mov Disord.

[2] Lwin A, Orvisky E, Goker-Alpan O, LaMarca ME, Sidransky E (2004). Glucocerebrosidase mutations in subjects with parkinsonism. Mol Genet Metab.

[3] Sidransky E, Nalls MA, Aasly JO, Aharon-Peretz J, Annesi G, Barbosa ER (2009). Multicenter analysis of glucocerebrosidase mutations in Parkinson’s disease. N Engl J Med.

[4] Aharon-Peretz J, Rosenbaum H, Gershoni-Baruch R (2004). Mutations in the glucocerebrosidase gene and Parkinson’s disease in Ashkenazi Jews. N Engl J Med.

[5] Mazzulli JR, Xu YH, Sun Y, Knight AL, McLean PJ, Caldwell GA (2011). Gaucher disease glucocerebrosidase and α-synuclein form a bidirectional pathogenic loop in synucleinopathies. Cell.

[6] Gegg ME, Burke D, Heales SJR, Cooper JM, Hardy J, Wood NW (2012). Glucocerebrosidase deficiency in substantia nigra of parkinson disease brains. Ann Neurol.

[7] Murphy KE, Gysbers AM, Abbott SK, Tayebi N, Kim WS, Sidransky E (2014). Reduced glucocerebrosidase is associated with increased alpha-synuclein in sporadic Parkinson’s disease. Brain.

[8] Persichetti E, Chiasserini D, Parnetti L, Eusebi P, Paciotti S, De Carlo C (2014). Factors influencing the measurement of lysosomal enzymes activity in human cerebrospinal fluid. PLoS One.

[9] Balducci C, Pierguidi L, Persichetti E, Parnetti L, Sbaragli M, Tassi C (2007). Lysosomal hydrolases in cerebrospinal fluid from subjects with Parkinson’s disease. Mov Disord.

[10] Parnetti L, Balducci C, Pierguidi L, De Carlo C, Peducci M, D’Amore C (2009). Cerebrospinal fluid beta-glucocerebrosidase activity is reduced in Dementia with Lewy Bodies. Neurobiol Dis.

[11] Parnetti L, Chiasserini D, Persichetti E, Eusebi P, Varghese S, Qureshi MM (2014). Cerebrospinal fluid lysosomal enzymes and α-synuclein in Parkinson’s disease. Mov Disord.

[12] McNeill A, Magalhaes J, Shen C, Chau KY, Hughes D, Mehta A (2014). Ambroxol improves lysosomal biochemistry in glucocerebrosidase mutation-linked Parkinson disease cells. Brain.

[13] Van Dijk KD, Persichetti E, Chiasserini D, Eusebi P, Beccari T, Calabresi P (2013). Changes in endolysosomal enzyme activities in cerebrospinal fluid of patients with Parkinson’s disease. Mov Disord.

[14] Nalls MA, Duran R, Lopez G, Kurzawa-Akanbi M, McKeith IG, Chinnery PF (2013). A multicenter study of glucocerebrosidase mutations in dementia with Lewy bodies. JAMA Neurol.

[15] Kurzawa-Akanbi M, Hanson PS, Blain PG, Lett DJ, McKeith IG, Chinnery PF (2012). Glucocerebrosidase Mutations alter the endoplasmic reticulum and lysosomes in Lewy body disease. J Neurochem.

[16] Murrey HE, Hsieh-Wilson LC (2008). The chemical neurobiology of carbohydrates. Chem Rev.

[17] Olanow CW (2007). The pathogenesis of cell death in Parkinson’s disease–2007. Mov Disord.

[18] Bosco DA, Fowler DM, Zhang Q, Nieva J, Powers ET, Wentworth P (2006). Elevated levels of oxidized cholesterol metabolites in Lewy body disease brains accelerate alpha-synuclein fibrilization. Nat Chem Biol.

[19] Cleeter MWJ, Chau KY, Gluck C, Mehta A, Hughes DA, Duchen M (2013). Glucocerebrosidase inhibition causes mitochondrial dysfunction and free radical damage. Neurochem Int.

[20] Ginns EI, Mak SK-K, Ko N, Karlgren J, Akbarian S, Chou VP (2014). Neuroinflammation and α-synuclein accumulation in response to glucocerebrosidase deficiency are accompanied by synaptic dysfunction. Mol Genet Metab.

[21] Reeve AK, Park T-K, Jaros E, Campbell GR, Lax NZ, Hepplewhite PD (2012). Relationship between mitochondria and α-synuclein: a study of single substantia nigra neurons. Arch Neurol.

[22] Schmittgen TD, Livak KJ (2008). Analyzing real-time PCR data by the comparative CT method. Nat Protoc.

[23] R Core Team (2013). R: A language and environment for statistical computing.

