# Supplementary material for: Selective loss of glucocerebrosidase activity in sporadic Parkinson’s disease and dementia with Lewy bodies
Source: Mol Neurodegener. 2015 Mar 27;10:15. doi: 10.1186/s13024-015-0010-2 (PMC4428238; doi:10.1186/s13024-015-0010-2)
Supplement: Additional file 1: Figure S1. — Boxplot of lysosomal enzyme activities significantly different among the experimental groups. Table S1. Lysosomal enzymes activities for brain areas and groups. [file 13024_2015_10_MOESM1_ESM.docx]

**Supplementary material**

**Supplementary figure 1**

**
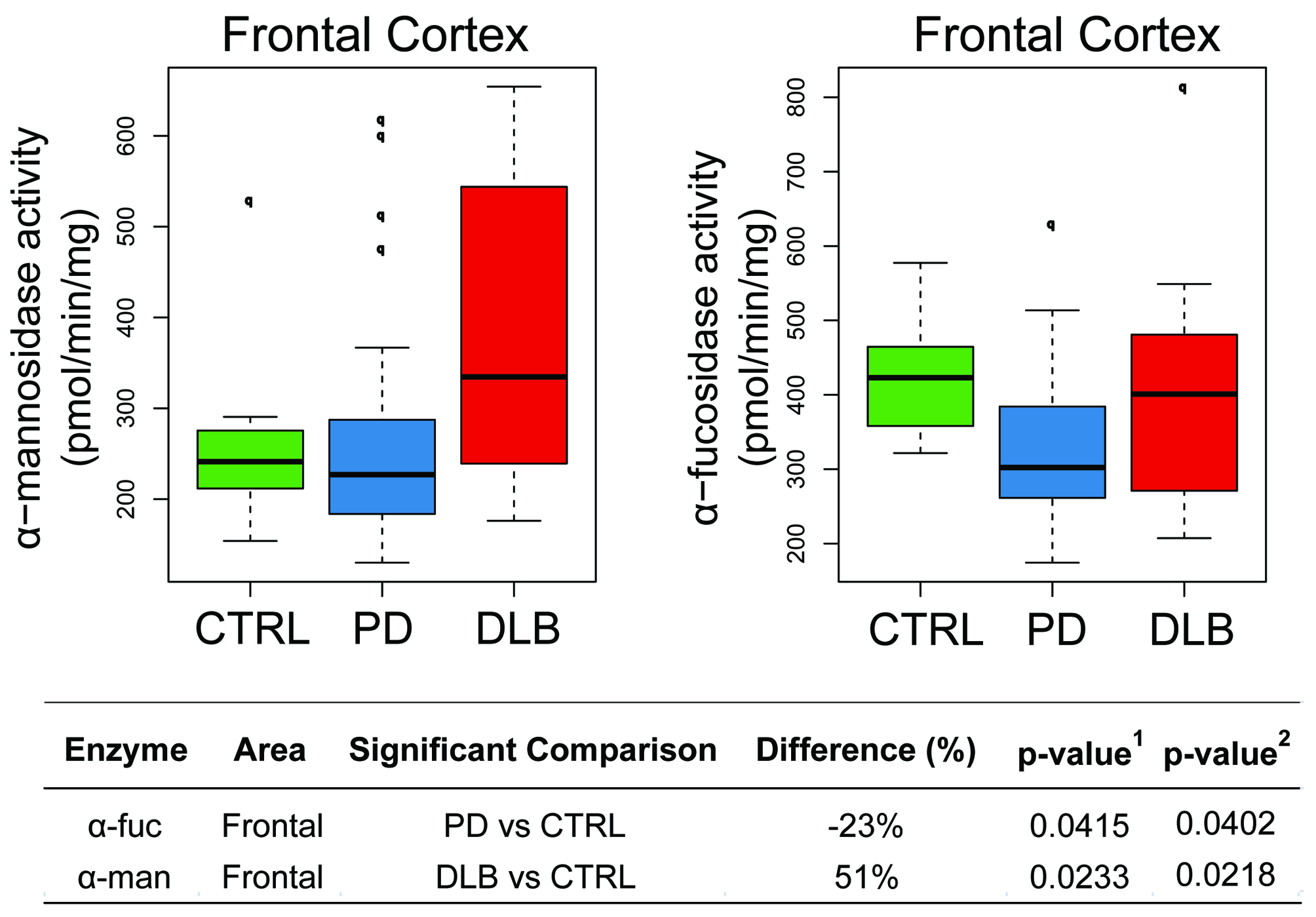
**

Boxplot of lysosomal enzyme activities significantly different among the experimental groups. Alpha-mannosidase (α-man) activity was significantly increased in DLB patients with respect to control subjects in the frontal cortex, while α-fucosidase (α-fuc) activity was decreased in the same brain area in PD patients with respect to controls. ^1^ Linear model with contrasts PD vs CTRL and DLB vs CTRL ^2^ Linear model with contrasts PD vs CTRL and DLB vs CTRL, with an adjustment for post-mortem delay.

**Supplementary Table 1** - Lysosomal enzymes activities for brain areas and groups.

|  |  | **CTRL** | **PD** | **DLB** |
| --- | --- | --- | --- | --- |
| **β-hexosaminidase** | **Caudate** | 9406.66 (±2197.27), 6283.32-13204.73 | 9019.96 (±2921.75), 4676.41-15322.57 | 8637.07 (±2351.37), 5151.09-13368.51 |
|  | **Cerebellum** | 12207.37 (±2905.11), 8072.02-17095.56 | 9302 (±4036.25), 2108.85-17562.15 | - |
|  | **Frontal Cortex** | 10195.99 (±1912.51), 6775.24-12862.22 | 9797.47 (±2250.34), 6763.48-17423.7 | 9673.68 (±2315.2), 5804.45-13919.96 |
|  | **Hippocampus** | 12494.52 (±2013.57), 8818.31-15583.93 | 12882.02 (±2895.87), 8897.4-19240.38 | - |
|  | **Putamen** | 13752.79 (±2093.79), 10088.48-16985.04 | 13446.64 (±2077.83), 10573.18-17863.25 | - |
|  | **Substantia Nigra** | 11455.54 (±1081.88), 10374.46-13520.86 | 11973.26 (±1997.36), 8693.98-15592.9 | 12004.75 (±2419.5), 9882.34-16428.25 |
| **α-fucosidase** | **Caudate** | 339.03 (±67.36), 202.38-432.81 | 296.5 (±138.63), 123.4-748.01 | 292.96 (±102.01), 155.31-485.37 |
|  | **Cerebellum** | 346.04 (±100.68), 226.41-517.86 | 261.91 (±78.15), 157.62-398.71 | - |
|  | **Frontal Cortex** | 425.7 (±88.78), 321.62-577.31 | **329.38 (±104.91), 174.28-630.32*** | 394.63 (±158.4), 207.16-814.29 |
|  | **Hippocampus** | 690.76 (±289.6), 437.75-1305.03 | 626.81 (±295.42), 223.73-1158.23 | - |
|  | **Putamen** | 508.65 (±114.55), 378.49-707.72 | 409.67 (±117.07), 264.82-637.68 | - |
|  | **Substantia Nigra** | 628.41 (±86.49), 500.8-749.61 | 537.63 (±144.25), 253.62-755.97 | 632.31 (±239.45), 376.15-1030.69 |
| **β-mannosidase** | **Caudate** | 164.66 (±36.26), 111.5-238.32 | 163.39 (±69.65), 44.34-357.46 | 187.64 (±72.86), 69.24-389.99 |
|  | **Cerebellum** | 371.5 (±133.75), 200.51-564.47 | 307.32 (±127.61), 148.8-599.41 | - |
|  | **Frontal Cortex** | 198.85 (±44.15), 144.83-302.09 | 223.08 (±133.7), 87.34-567.17 | 282.91 (±92.81), 146.92-450.05 |
|  | **Hippocampus** | 327.97 (±36.55), 277.41-389.72 | 415.03 (±172.12), 232.54-758.56 | - |
|  | **Putamen** | 290.94 (±76.07), 162.38-378.85 | 250.75 (±80), 139.37-360.01 | - |
|  | **Substantia Nigra** | 539.35 (±25.27), 511.08-574.81 | 724.09 (±258.56), 325.58-1126.43 | 655.36 (±230.16), 402.53-1015.81) |
| **α-mannosidase** | **Caudate** | 191.45 (±40.38), 120.29-269.16 | 199.23 (±122.04), 59.03-446.4 | 182.89 (±63.68), 104.08-298.17 |
|  | **Cerebellum** | 219.78 (±42.2), 166.35-267.93 | 245.39 (±66.62), 143.38-362.26 | - |
|  | **Frontal Cortex cortexCortex** | 259.54 (±103.65), 154.01-529.43 | 276.17 (±138.18), 130.05-618.49 | **392.49 (±162.03), 176.2-654.09*** |
|  | **Hippocampus** | 367.57 (±66.91), 276.45-454.9 | 415.07 (±83.88), 288.32-546.74 | - |
|  | **Putamen** | 283.8 (±92.68), 178.22-534.76 | 240.08 (±66.9), 149.28-369.09 | - |
|  | **Substantia Nigra** | 408.13 (±102.79), 294.82-507.47 | 416.97 (±110.14), 128.67-568.36 | 452.25 (±90.25), 348.16-586.8 |
| **β-galactosidase** | **Caudate** | 501.27 (±161.97), 85.62-695.9 | 499.48 (±160.72), 231.9-763.64 | 521.3 (±115.99), 336.16-806.16 |
|  | **Cerebellum** | 875.3 (±238.24), 563.87-1236.18 | 809.09 (±180.26), 503.85-1086.27 | - |
|  | **Frontal Cortex** | 631.05 (±152.68), 399.21-879.59 | 650.69 (±153.41), 340.68-953.04 | 655.84 (±127.83), 430.72-820.7 |
|  | **Hippocampus** | 813.99 (±185.48), 588.37-1115.09 | 861.93 (±346.53), 353.42-1618.98 | - |
|  | **Putamen** | 721.13 (±146.46), 460.31-928.24 | 744.29 (±162.57), 565.54-1075.14 | - |
|  | **Substantia Nigra** | 890.47 (±154.78), 655.63-1083.17 | 794.11 (±193.81), 502.27-1228.49 | 887.48 (±137.01), 738.7-1074.55 |
| **GCase** | **Caudate** | 561.94 (±101.73), 292.48-668.52 | **452.08 (±152.12), 210.65-747.83*** | 496.44 (±95.61), 279.94-639.36 |
|  | **Cerebellum** | 692.24 (±123.78), 467.33-854.09 | 671 (±189.08), 405.28-1071.76 | - |
|  | **Frontal Cortex** | 740.3 (±140.81), 463.13-923.55 | 718.46 (±198.21), 353.1-1020.98 | 630.5 (±137.82), 424.82-862.37 |
|  | **Hippocampus** | 690.8 (±124.76), 530.91-911.96 | 769.68 (±230.55), 416.11-1084.02 | - |
|  | **Putamen** | 683.51 (±78.86), 555.73-774.5 | 684.71 (±194.82), 333.99-1023.13 | - |
|  | **Substantia Nigra** | 727.91 (±60.61), 648.72-799.45 | **626.79 (±52.73), 532.44-706.39*** | 639.76 (±150.26), 460.52-901.76 |
| **Cathepsin E** | **Caudate** | 28.31 (±13.5), 11.38-48.5 | 30.80 (±20.98), 3.26-84.87 | 31.16 (±13.68), 15.09-58.34 |
|  | **Cerebellum** | 75.99 (±13.06), 63.81-96.47 | 74.57 (±18.73), 48.19-109.79 | - |
|  | **Frontal Cortex** | 38.42 (±10.15), 26.23-52.22 | 39.06 (±10.80), 23.55-67.07 | 39.85 (±16.38), 17.62-68.61 |
|  | **Hippocampus** | 47.68 (±6.71), 37.33-55.87 | 54.54 (±10.39), 37.47-72.78 | - |
|  | **Putamen** | 43.78 (±14.56), 25.53-73.42 | 38.64 (±16.56), 16.7-69.08 | - |
|  | **Substantia Nigra** | 76.71 (±12.66), 64.67-94.71 | 74.35 (±12.65), 53.99-89.66 | 69.83 (±9.72), 57.94-81.86 |

Means (±SD), (min-max). *p<0.05 with respect to control group. All data are reported in pmol/min/mg of proteins.
